# Neglected and lethal: Case series of fatal scrub typhus in Malaysia

**DOI:** 10.1371/journal.pntd.0013156

**Published:** 2025-12-23

**Authors:** Siti Roszilawati Ramli, Shirley Yi Fen Hii, Suhaili Zainal Abidin, Kok Soon Lee, Tiang Koi Ng, Nor Arisah Misnan, Mohammad Yazid Abdad

**Affiliations:** 1 Bacteriology Unit, Infectious Disease Research Centre, Institute for Medical Research, National Institute of Health, Shah Alam, Selangor, Malaysia; 2 Acarology Unit, Infectious Disease Research Centre, Institute for Medical Research, National Institute of Health, Shah Alam, Selangor, Malaysia; 3 Internal Medicine Department, Hospital Tuanku Ja’afar, Seremban, Negeri Sembilan, Malaysia; 4 Internal Medicine Department, Hospital Sungai Buloh, Sungai Buloh, Selangor, Malaysia; 5 Internal Medicine Department, Hospital Shah Alam, Shah Alam, Selangor, Malaysia; 6 Mahidol Oxford Tropical Medicine Research Unit, Faculty of Tropical Medicine, Mahidol University, Bangkok, Thailand; 7 Nuffield Department of Clinical Medicine, Centre for Tropical Medicine, John Radcliffe Hospital, Oxford, United Kingdom; Yale University School of Medicine, UNITED STATES OF AMERICA

## Abstract

Scrub typhus (ST), an often-underdiagnosed zoonotic infection, poses significant diagnostic and management challenges, particularly in regions with endemic tropical infections like Malaysia. We report three fatal cases of ST in central region West Malaysia, highlighting diagnostic pitfalls and implications for clinical management. Three fatal cases of ST presented with respiratory distress and multiorgan failure were reported in this study. Laboratory investigations confirmed by positive *Orientia tsutsugamushi* (OT) PCR results. All three cases exhibited low PCR cycle threshold (Ct) values, suggesting high bacterial loads linked to severe disease. Phylogenetic analysis indicated two distinct genotypes, underscoring diverse circulating strains in Malaysia. Eschars were present but not initially detected in two cases. These cases emphasize critical diagnostic challenges in ST due to its nonspecific presentation and reliance on clinical recognition of eschar. Early identification and molecular confirmation are vital to improve diagnostic accuracy and reduce morbidity and mortality of ST.

## Author summary

Scrub typhus (ST), an endemic zoonotic disease in Malaysia since its first probable report in 1915, has shown a concerning resurgence in recent years. Contributing factors include climate change, agricultural expansion into rural areas, and improved diagnostic capabilities. This report presents three recent fatal cases of ST in Malaysia, underscoring its continued threat to public health. Case 1 involved a 48-year-old Indonesian rubber tapper who presented with severe sepsis and respiratory failure. Despite intensive care and empirical treatment, he died within 24 hours. Case 2 was a 33-year-old female lemongrass farm worker with septic shock and multiorgan failure, who had a misdiagnosis of dengue and leptospirosis before eschar observation led to ST confirmation via PCR. Despite targeted therapy, she deteriorated rapidly. Case 3 was a 47-year-old man with comorbidities who presented late with respiratory distress and renal failure and succumbed to refractory shock despite broad spectrum antibiotics coverage. Case 1 and 2 admitted after onset of illness of 1 week duration while Case 3 was hospitalized after 2 weeks of disease onset. An eschar was discovered late in both Case 2 and 3 which led to the eventual diagnosis of ST. These cases highlight diagnostic challenges, frequent misclassification as other endemic febrile illnesses, and the urgency for heightened clinical awareness and surveillance to mitigate ST-related mortality in Malaysia.

## Introduction

Malaysia is no stranger to ST, with the first probable case reported as far back as 1915, described as pseudo-typhoid and carried by trombiculid mites. Since then, more evidence and reports have further described ST infections in Malaysia, with acceptance of its endemicity in both East and West Malaysia. With the advent of modern antibiotics, many bacterial diseases have become easily treatable. However, the re-emergence of ST in Malaysia has been observed to be on the upswing. This could be caused by a combination of various factors such as climate change, expansion of farming lands and activities into undisturbed rural areas, and more accurate and highly accessible diagnostic tools. We present here three recent fatal cases of ST who contracted the infection in the state of Negeri Sembilan, further highlighting the threat that ST continue to pose to the public health of Malaysia as a re-emerging disease, well into the 21^st^ century.

### Case 1

A 48-years-old male migrant worker from Indonesia, with no known medical illness presented with fever for 1 week duration associated with chills and rigors, arthralgia, myalgia and headache. He also had poor appetite followed by persistent vomiting & diarrhoea. He appeared lethargic in the last few days. There was no haematemesis, bloody diarrhoea, rashes, bruises, bleeding tendency nor other relevant symptoms. He had multiple visits to the primary healthcare facilities and was given symptomatic treatment. Upon arrival at Emergency Department HTJ Seremban, Negeri Sembilan he was restless, confused, cyanosed, and marked rapid breathing. Oxygen saturation was 70% under high flow mask oxygen (HFM O2), required immediate intubation and mechanical ventilation. His blood pressure was 69/53 mmHg preintubation, which required fluid resuscitation and inotropic support. His pulse rate was 196 beats per minutes with poor pulse volume and capillary refill time was more than 2 seconds with coolish peripherals. Other systemic examinations were unremarkable. He was a rubber tapper in Simpang Durian, Jelebu of the state of Negeri Sembilan for more than 10 years, and no recent history of travelling. The patient was reported as a chronic smoker. There was no recent fogging or known dengue cases in the area. He lived with his friends but they were asymptomatic.

Amoxicillin-clavulanic acid and doxycycline were prescribed and immediately admitted to ICU. The arterial blood gas (HFM O_2_) results initially showed severe over compensated metabolic acidosis: pH 7.55., PCO_2_ 9.4 mmHg, PO_2_ 98.4 mmHg, HCO_3-_ 8.4 mEq/L, but repeated blood gases post intubation: pH 7.397, PCO_2_ 19.1 mmHg, PO_2_ 394.2 mmHg, HCO_3-_ 11.8 mEq/L. Electrocardiography revealed supraventricular tachycardic (SVT) initially and synchronised cardioverted to sinus tachycardic. Creatinine kinase was 781 (unit), troponin I negative. Dextrostix reading was 7.0 mmol/L. Human immunodeficiency virus (HIV) antigen/ antibody (Ag/Ab), Hepatitis surface antigen (HBsAg), Anti Hepatitis C virus antibody, rapid plasma reagins (RPR) test to screen for syphilis was non-reactive. CXR normal. Bedside echocardiogram was hyperdynamic heart contractility, no pericardial effusion. His condition quickly deteriorated and succumbed within 24 hours of admission to the intensive care unit (ICU).

### Case 2

Subject is a 33-year-old female who presented with fever for one week, associated with vomiting, loose stool, and chest discomfort. She had a history of a visit to a health clinic and was treated with antibiotics but symptoms did not abate. She was a worker in a lemongrass farm near district of Baranang with potential exposure to rodents. She arrived at the emergency department critically ill, was admitted to ICU and electively intubated due to respiratory failure. Patient was empirically started on benzylpenicillin to cover for leptospirosis which was suspected earlier. Upon further assessment, there was presence of eschar near the subject’s inguinal region. Scrub typhus treatment including intravenous azithromycin and doxycycline were initiated. After two days in the intensive care unit (ICU) the patient deteriorated further and passed on. Subject remained unwell in ICU, requiring inotropic support for septic shock and developed fast atrial fibrillation was electrically cardioverted three times and loaded with infusion amiodarone. Her kidney function also deteriorated with persistent metabolic acidosis. Antibiotics escalated to piperacillin- tazobactam. Patient was deemed clinical unstable for haemodialysis and hypotension worsened requiring increasing inotropic support. Subject unfortunately passed on in the ICU.

**Fig 1:**
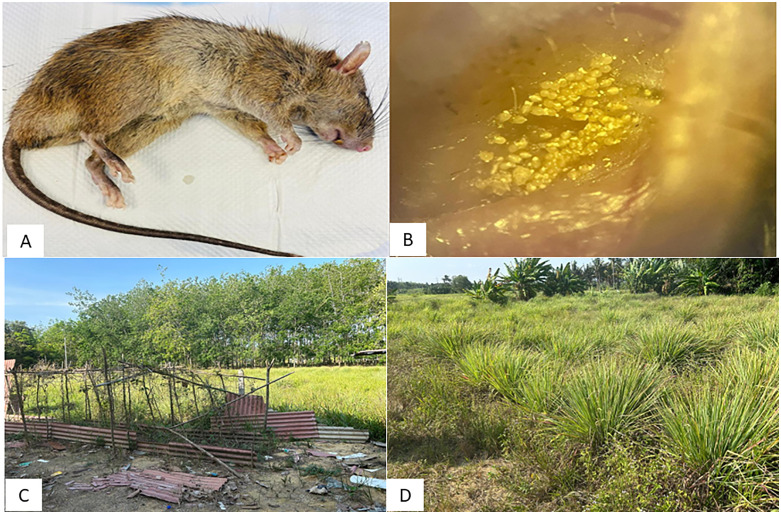
(A) *Rattus rattus* infested with chiggers trapped from one of the main lemongrass farms in Baranang. (B) Visualization of chiggers in the ear of a rodent at 40X microscope magnification. (C) The surrounding area of patient’s house (D) Lemongrass field where patients work in Baranang districts, Selangor.

Following the notification of this case to the Hulu Langat District Health Office, a post-diagnostic vector surveillance was immediately conducted at patient’s residence and workplace in Baranang, Selangor. Chiggers collected from the rodent were submitted to the Acarology Unit, Institute for Medical Research for molecular screening of OT (Fig 1).

### Case 3

Forty-seven year old male with known cases of hypertension and dyslipidemia presented to the emergency department at HTJ with respiratory failure type 1 evidenced by shortness of breath and tachycardia. He was admitted to a high dependency unit, intubated and supported with inotropes. Two weeks prior to admission he had a complaint of intermittent fever followed by a 1-week history of vomiting, diarrhoea and abdominal pain. He went to two general practitioners and was prescribed antibiotics and referral to hospitals however was deferred. He was a businessman working in Lenggeng, Negeri Sembilan and under follow-up at Lenggeng Health Clinic. The patient worked at a construction site and from home, and reportedly took care of stray cats at home and adopted more than 10 cats.

**Fig 2:**
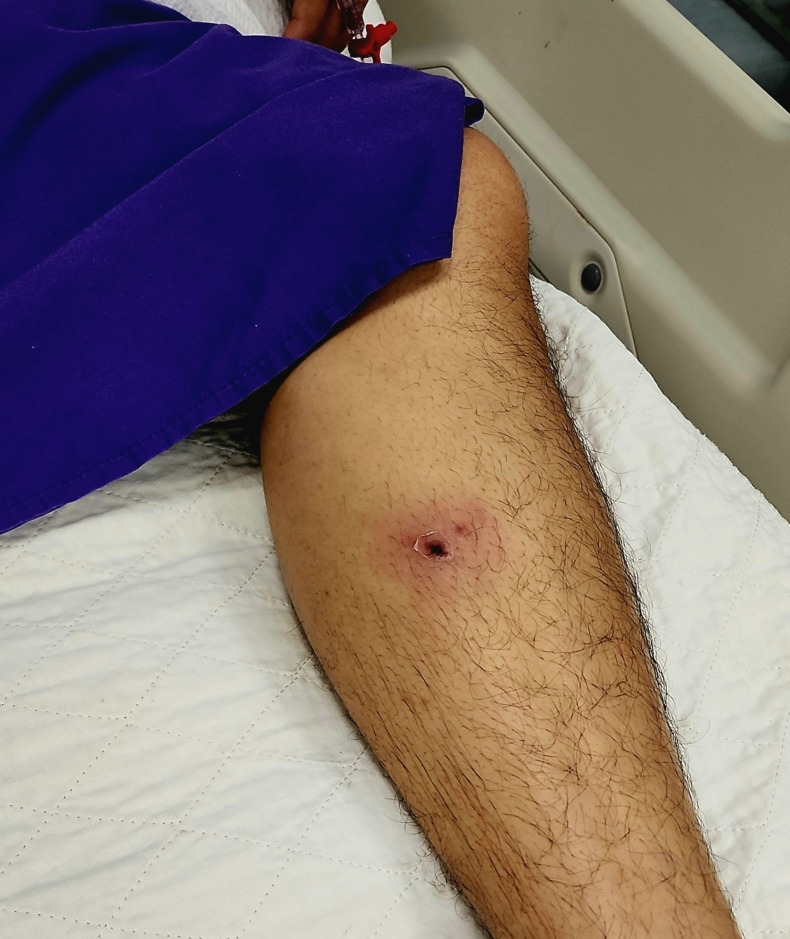
Eschar on left inner shin in Case 3.

During admission, he developed acute kidney failure requiring sled and transfusion. On the 4th day of admission, a visiting Infectious Disease specialist in ICU noted an eschar on the left inner shin with erythematous borders (Fig 2). There was no further history received from the family regarding the skin lesion. He also had few episodes of passing out melenic stools. He was diagnosed with multifactorial shock in disseminated intravascular coagulopathy (DIVC). First possible cause was septic shocks secondary to infective acute gastrointestinal; presumed leptospirosis with multiorgan involvement and acute kidney injury with transaminitis and myositis. Second possible cause was hypovolemic shock secondary to gastrointestinal bleeding. Patient succumbed due to refractory shock with DIVC two days after the biopsy despite empirical therapy of ceftriaxone, doxycycline and azithromycin.

Standard blood investigations were performed on all three cases which include full blood count, liver function test, renal profile, C-reactive protein. Specific routine tests for tropical febrile illnesses, i.e., blood film malaria parasite, dengue Combo test NS1, IgM and IgG serology, dengue PCR, leptospirosis IgM and PCR test performed based on methods described in previous report [1–4]. Blood and eschar biopsy were sent for Rickettsial PCR (which detects *Rickettsia* spp and *Orientia tsutsugamushi* (OT)) performed at Rickettsial reference laboratory. Institute for Medical Research, Setia Alam, Selangor.

## Methods

### Ethics statement

This study was registered with the National Medical Research Register (NMRR) and ethically approved by the Medical Research and Ethics Committee (MREC), Ministry of Health, Malaysia with reference number NMRR-24–02585-ZD9 on 2nd October 2024. All samples included in this study were post-diagnostic specimens collected as part of routine clinical care and processed according to standard protocols. Prior to inclusion in the study, all samples were de-identified and anonymized to ensure patient confidentiality. As such, the requirement for informed consent was waived.

### DNA extraction from patient blood and chigger samples

#### Patient blood samples.

The blood samples of the three patients were sent in EDTA tubes to Bacteriology unit, Institute for Medical Research which serves as the reference laboratory for Rickettsial diseases in Malaysia. The blood samples were centrifuged at 4000 rpm for 10 minutes to separate the blood layers to obtain the buffy coat. 300 µl of the buffy coat were collected and proceed with DNA extraction by using QIAmp DNA Blood Mini Kit (Qiagen, Germany). DNA was eluted in 50 µl AE elution buffer and stored in -40^o^C until use.

#### Chigger samples.

The ethanol-preserved chiggers were pooled into groups (*n* = 15 per pool) and were rinsed with distilled water for five times. Whole chiggers were homogenized using sterile micropestles in ATL buffer with added proteinase K and incubated overnight at 56°C. Genomic DNA was extracted using the QIAamp DNA Mini Kit (Qiagen, Germany) according to the manufacturer’s protocol. DNA was eluted in 50 µL AE buffer and stored at –20°C.

## Detection and genotyping of orientia tsutsugamushi DNA

### Patient blood samples

Genomic DNA extracted from buffy coat were first screened for the presence of OT by real-time PCR assay targeting the 47kDa periplasmic serine protease *htrA* (47 kDa) gene using the primers and probe as described by Jiang et al., 2004 [5]. The procedure was performed using 4X CAPITAL qPCR Probe Master Mix (Biotech Rabbit, Germany) according to the manufacturer’s protocol. Briefly, a total of 25 µl reaction per tube was prepared including 6.25 µl master mix and 1 µl of each primer and probe. The two-step qPCR reaction was performed on QuantStudio 6 FlexS Real-Time PCR system (Thermo Fisher Scientific, USA) at initial denaturation:94 ^o^C for 5 minutes followed by 40 cycles of denaturation at 94 °C, 5 sec, and annealing at 60 °C, 30 sec.

The DNA were later proceeded for nested PCR to determine the genotypes of the OT strains. Briefly, nested OT PCR was used to amplify outer and inner fragments of *tsa56* using the inner and outer primer sets [6,7]. The first amplification mixture (50 µl per reaction) started with a 5 µl template DNA to amplify the outer primers fragments using PCRBIO Ultra mix (PCR Biosystems, USA). For the second PCR, 5 µl of the first PCR was used as template to amplify the inner primers fragments. The PCR conditions were the same for both first and second PCR: initial denaturation at 94 °C for 2 sec, denaturation at 94 °C for 30 sec, annealing at 57 °C for one min and extension at 72 °C for one min for 35 cycles and final extension at 72 °C for 10 min. The PCR product was run on a 2% agarose gel and viewed on Bio-Rad ChemiDoc Touch Image System (Bio-Rad, USA). The final PCR products (inner fragments of *tsa56* gene) was sequenced by Sanger sequencing (Apical Scientific, Malaysia). the three *tsa56* gene sequences were aligned together with other OT genotype strains from the GeneBank and tree was generated using the Maximum likelihood method conducted in MEGA 11 [8]. The tree is generated by bootstrap analysis with 1000 repetitions to assess the reliability of the tree branching. The partial *tsa56* sequences of IMRS_RE615, IMRS_RE282 and IMRS_RE451 have been deposited in GenBank (PV948461-PV948463).

### Chigger samples

Pooled chigger DNA was screened using nested PCR targeting the 56-kDa type-specific antigen (*tsa56*) gene of OT, following the protocol by Furuya et al. [9]. The PCR was performed using MyTaq HS Red Mix (Bioline, UK) in a 25 µL reaction volume. Each 25 µL PCR reaction contained 1 × PCR buffer, 1.5 mM MgCl₂, 200 µM dNTPs, 0.4 µM of each primer, 1.25 U Taq DNA polymerase (Thermo Fisher Scientific), and 2 µL of template DNA. Thermal cycling consisted of an initial denaturation at 94°C for 1 minutes; 35 cycles of denaturation at 94°C for 15 seconds, annealing at 57°C for 15 seconds, extension at 72°C for 10 seconds. Amplicons were separated on a 1.5% agarose gel stained with ethidium bromide and visualized using a Bio-Rad ChemiDoc Touch Imaging System. One positive pool for OT was purified using the QIAquick PCR Purification Kit (Qiagen) and sequenced by Sanger method (Apical Scientific, Malaysia).

## Results

All cases showed positive Rickettsia PCR with band detected on nested PCR *tsa* gene 56kDA. The results of 47 kDA showed that all three fatal cases exhibited low cycle threshold (Ct) values, indicative of a high bacterial load, with Ct values of 30, 27, and 25 for Case 1, Case 2, and Case 3, respectively. Blood culture for Case 2 revealed growth of *Staphylococcus borealis* which was likely a contaminant. Urine and tracheal aspirate culture sent for Case 3 yielded no growth. Although Leptospira IgM rapid test result was positive, her Leptospirosis PCR result was negative, thus co-infection with Leptospirosis was unlikely and the qualitative serology result was false positive. All blood investigation results were summarised in Table 1.

**Table 1:** Summary of medical history, clinical features, blood investigations, treatment and outcome of the three case studies.

	Case 1–48/ M	Case 2–33/ F	Case 3–47/M
Admission	Hospital Tuanku Jaafar, Negeri Sembilan	Hospital Tuanku Jaafar, Negeri Sembilan	Hospital Shah Alam, Selangor
Onset	Fever 7 days +	Fever 7 days +	Fever 14 days
Signs & symptoms	SOB, chills, rigors, arthalgia, myalgia, headache, LOA, vomiting, diarrhea	SOB, Vomiting, loose stool, and chest discomfort,	SOB, vomiting, diarrhoea and abdominal pain, melenic stool,
Presence of eschar	No	Yes (left inguinal)	Yes (Left inner shin)
Medical history	N/A	N/A	Hypertension, dyslipidemia
Exposure history	Rubber tapper at Jelebu No history of travelling to Indonesia	Lemongrass farmer at Baranang	Contractor at Lenggeng Take care of stray cats
White cell count (10^3^/µL)	15.2	21.5	27
White cell count (%)	Neutrophils 70.4%, Lymphocytes 17.1%	Neutrophils 88.0%, Lymphocytes 7.2%	N/A
Platelet count (10^3^/µL)	192	75	165-218
C-reactive protein (mg/L)	161.79	234	108
Alanine transaminase Aspartate transaminase (µg/L)	ALT 111 AST 113	ALT 74 AST 201	ALT 114–128 AST 258
Renal profile (µg/L)	Urea 30.1 Na + 125 K + 4.5 Creatinine	Urea 12.4 Na + 149 K + 4.7 Creatinine 209	Urea 596 Na + 127–132 K + 4.2- 4.4 Creatinine 680–1017
Blood Culture	No growth	*Staphylococcus borealis*	No growth
Rapid test kit – Dengue Combo test – NS1, IgM and IgG	Dengue Combo - negative NS1- negative IgM -negative IgG- negative	Dengue Combo: NS1- negative IgM - positive IgG - negative	Dengue Combo – not done
Rapid test kit – Leptospira IgM antibody	Negative	Positive	Inconclusive
Blood film malaria parasite	Negative	Negative	Negative
Dengue PCR	Dengue virus- not detected	Dengue virus - not detected	Dengue virus- not detected
Leptospirosis PCR	Leptospira *spp*- not detected	Leptospira *spp*- not detected	Leptospira *spp*- not detected
Rickettsioses PCR	OT PCR- DNA detected Rickettsia *spp* PCR- DNA not detected	OT PCR- DNA detected Rickettsia *spp* PCR *-* DNA not detected	OT PCR- DNA detected Rickettsia *spp* PCR *-*DNA not detected
Ct value of OT PCR	30 cycles	27 cycles	25 cycles
Treatment	Amoxycillin & Doxycyclin	Doxycyclin, Azythromycin, Pip-tazo	Ceftriaxone, Doxycyclin, Azithromycin
Expire	Within 24 hours after admission	3 days after admission	6 days after admission

SOB, shortness of breath; LOA, loss of appetite; LOW; loss of weight; ALT, Alanine transaminase; AST, Aspartate transaminase; Na + ,

sodium; K + , potassium; NS1, Non-structural protein 1; IgM, Immunoglobulin M; IgG, Immunoglobulin G; OT, *Orientia tsutsugamushi;*

Ct value; cycle threshold.

Genotyping of OT was based on the aligned consensus 480 bp of 88 *tsa56* sequences which consists of 4 local isolates and 84 isolates from other countries (S1 Table). The sequenced partial *tsa56* genes of IMRS_RE615, IMRS_RE451, IMRS_RE282 and Malaysian chiggers were first blasted to assess the completeness and the CDS region. The blast results were also used as reference for selection of isolates to be included in the phylogenetic analysis. Maximum likelihood tree was generated to allow comparison and groupings as suggested by Kim et al. [9]. The results showed that the human OT cases: IMRS_RE615 (Case 3) belongs to KarpA whilst IMRS_RE451 (Case 2) and IMRS_RE282 (Case 1) in TA763 group but sub-grouped as TA763B and TA763A, respectively (Fig 4).

**Fig 3:**
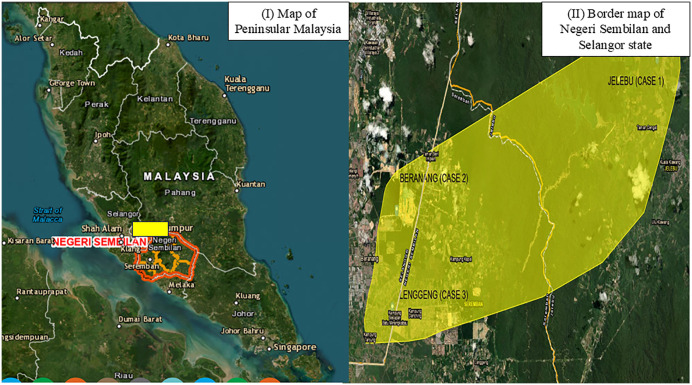
(I) Map of Peninsular Malaysia. Red outline indicate Negeri Sembilan state and yellow rectangle indicate the location of all three fatal ST cases. (II) Map of border between Negeri Sembilan and Selangor state. JELEBU (CASE 1) indicates the location to Case 1, while BERANANG (CASE 2) and LENGGENG (CASE 3) represent the location of exposure for Case 2 and 3 respectively. Images modified from: https://gis9.ns.gov.my/arcgisportal/apps/webappviewer/index.html?id=7a319cbeff7c4b9a9f2dfc569580db13 (accessed on 14^th^ December 2025).

Genotyping of OT based on the tsa56 sequences revealed that Case 1 and 2 were clustered together and highly related to TA763B strain, while Case 3 showed high similarity to the KarpA strain (Fig 3). Chiggers collected from *Rattus rattus* were also identified as belonging to the KarpA strain, though this did not correlate with the findings in Case 2. These results suggest the presence of more than one circulating strain of OT in the Baranang district.

All four cases were analysed together with *tsa56* sequences of various OT genotypes which includes Karp group (KarpA, B, C, Saitama, Boryong), TA763 group, Kato group (A, B), Gilliam group (GilA, GilB, GilC, Kawasaki), and Shimokoshi group [10].

## Discussion

Scrub typhus infection is one of the main causes of acute undifferentiated fevers in Southeast Asia. The clinical picture consists of abrupt onset fever with chills and non-specific symptoms such as headache, myalgia, and vomiting. Eschar could be considered as the most important clinical finding for the diagnosis of ST. The presence of an eschar, particularly in the axilla, groin, or inguinal regions, can be a strong clinical clue for diagnosing ST [11]. The rates of eschar varied depending on the geographical regions. Reported rates of eschar may range from 46% to 92% in Thailand and the Indian subcontinent respectively [11–13]. This is possibly related to the different virulence, the load of pathogen and host immunity. Most physicians can readily diagnose acute ST when the characteristic eschar is present, leading to prompt initiation of appropriate antibiotic therapy. However, the presence of eschar may still be missed when there was no awareness and suspicion for ST. In Case 2 and 3, this key clinical sign was overlooked during the initial clinical examinations, likely due to limited awareness of ST. Consequently, both patients did not receive appropriate antibiotic treatment at the outset. Previous studies have reported that early treatment is important to improve patients’ outcome and reduce life-threatening complications [14]. The primary treatment involves antibiotics from the tetracycline class however macrolides may be used as alternatives. Varghese et al recommended that both doxycycline and azithromycin can be used in severe ST cases [15–18].

Previous studies have identified bacterial load as a significant factor contributing to mortality in ST. In the present study, all three cases demonstrated Ct values of ≤ 30 on quantitative PCR targeting the 47-kDa gene, indicative of high bacterial burden. Notably, Case 3, which had the lowest Ct value among the three, also presented with the most severe clinical complications. This observation aligns with findings by Taylor et al., who reported a correlation between higher bacterial load and increased mortality [19]. These findings suggest that low Ct values (≤ 30) on quantitative PCR with target gene such as 47-kDA may serve as a potential surrogate marker for disease severity. Further validation through well-designed case-control studies with adequate sample sizes is necessary to establish the prognostic value of this marker in clinical practice. In addition, a separate study evaluating predictors of fatal outcomes identified elevated APACHE II scores as an independent risk factor for mortality [20,21].

In high-burden ST country such as India, approximately one-third of hospitalized patients develop organ dysfunction, which may manifest as respiratory failure, circulatory shock, mild renal or hepatic impairment or central nervous system involvement. Hemorrhagic manifestations and coagulation disorders, particularly gastrointestinal complications, have also been reported among severely ill patients [22–26]. While recovery is generally complete in mild cases, severe disease with multi-organ failure is associated with mortality rates as high as 30% [27,28]. In this report , all cases exhibited respiratory failure, renal and hepatic impairment as well as septic shock. Nevertheless, Case 3 exhibited the most severe complications, i.e., renal failure, coagulation disorders, and upper gastrointestinal bleeding. In addition to pathogen factors such as strain virulence and bacterial inoculum, the presence of underlying medical conditions of hypertension and dyslipidemia as well as older age potentially reduce host immunity and complicates the response to infection which ultimately contributed to the higher disease severity [24].

A study by Taylor et al reported lower incidence of case fatality rate of ST among local populations compared to non-residents with no prior exposure to the disease [19]. Consistent with this observation, our first two cases supported this hypothesis: the first case involved an immigrant worker from Indonesia, while the second case originated from Sabah, East Malaysia. In contrast, the third case was a resident of Shah Alam, Selangor, which is geographically closer to the identified exposure districts, particularly Lenggeng in Negeri Sembilan. As illustrated in Fig 3, the exposure sites for all three cases are located in close proximity. Beranang, a district in Selangor, lies within a 5–40 km radius of Lenggeng and Jelebu—two districts in Negeri Sembilan. The border region between Selangor and Negeri Sembilan is hypothesized to be a potential hotspot for chigger infestation. Further studies are warranted through targeted chigger exploration and surveillance studies to better understand the vector distribution, circulating strains and disease transmission risk.

Based on the phylogenetic analysis in this study, there are two circulating genotypes in Negeri Sembilan state: Karp and TA763. Both IMRS_RE615 (Case 3) and Malaysian chiggers were of KarpA genotype. Despite being in the same group, both strains branched at different node. The strains showed clustering with Taiwan, China, India, Cambodia, Vietnam and Thailand. The different branching indicates that although both were of KarpA group, they might originate from different sources. Both IMRS_RE451 and IMRS_RE282 are grouped in the TA763 group albeit different subgroup. IMRS_RE451 (TA763B) was more closely related to strains from Taiwan and India compared to neighbours, Thailand and Vietnam. IMRS_RE282 (TA763A) was clustered together China and Taiwan. The differences could be due to geographical variation within TA763 groups as TA763A is more prevalent in China and Taiwan compared to TA763B [10]. In addition, both strains differed where IMRS_RE451 clustered with the strains isolated before year 2012 and IMRS_RE282 with more recent strains at year 2019 (S1 Table). However, we also observed that IMRS_RE282 and other TA763A strains shared the same node with TA763 isolated from rats in Thailand in year 1963, depicting that there is little genetic recombination or mutations occurring in TA763A strains over six decades. It was previously reported that the emerging of subgroups could be due to genetic recombination events occurring within the parental groups (Karp, Gilliam and Kato) [10].

Locationwise, Malaysia chigger strain was successfully sampled from rat in Beranang district, near to the location of Case 2 (IMRS_RE451), both were of different genotype: KarpA vs TA763B. However, it is to note that the distance between Beranang (Case 2, IMRS_RE451, TA763B) and Lenggeng (Case 3, IMRS_RE615, KarpA) were approximately 5 km whilst Jelebu (Case 1, IMRS_RE282, TA763A) is 34–40 km apart from both locations (based on Google Map distance). In this study, we observed fatality in Karp and TA763 groups. The pathogenicity and virulence of different OT genotypes is still unconvinced. There was evidence on Karp genotypes causes more severe outcome and significantly higher DNA load compared to the other genotypes [22,23].

Due to the nonspecific clinical manifestations of ST, which often mimic other endemic tropical diseases such as dengue, leptospirosis, malaria, and enteric fever, specific diagnostic testing is essential for accurate identification. Serological assays have been the mainstay of diagnosis prior to the advent of molecular methods. However, serology assays have several limitations, including variable sensitivity and specificity, cross-reactivity with other tropical infections, and delays in diagnosis due to the requirement for paired acute and convalescent samples to confirm recent infection. The introduction of molecular assays has significantly enhanced the accuracy and timeliness of ST diagnosis. In some regions, the increased detection of cases using molecular assays has led to the recognition of ST as a re-emerging infectious disease after a period of neglect [23–29].

The cases in this study highlight significant diagnostic challenges that hinder prompt treatment, particularly in settings where ST may not be immediately suspected. Misdiagnosis or delayed recognition can result in inadequate management, leading to severe complications, including organ failure and death, as seen in these cases. Overlapping signs and symptoms with other endemic tropical diseases, complicate clinical recognition, especially when eschar is absent or overlooked. Implementing awareness programs and diagnostic protocols that incorporate both molecular and serology testing could improve timely identification. The varied genotypes detected in these cases suggest a complex landscape of OT strains in Malaysia, which may contribute to differences in disease severity and clinical presentation. There is also a low number of isolates used in the genotyping study due to limited Malaysian OT TSA56 genes available in the public database. Despite the limitations, this study provides an insight into the possible presence of various OT genotypes in Malaysia, warranting a further study to initiate an on-going surveillance of the Malaysian OT genotypes that is critical to provide more information on the fatality level of circulating genotypes in Malaysia. Enhanced clinical awareness and a comprehensive One Health approach genomic surveillance on a region-specific basis could be pivotal for better case management and reducing mortality from ST.

**Fig 4:**
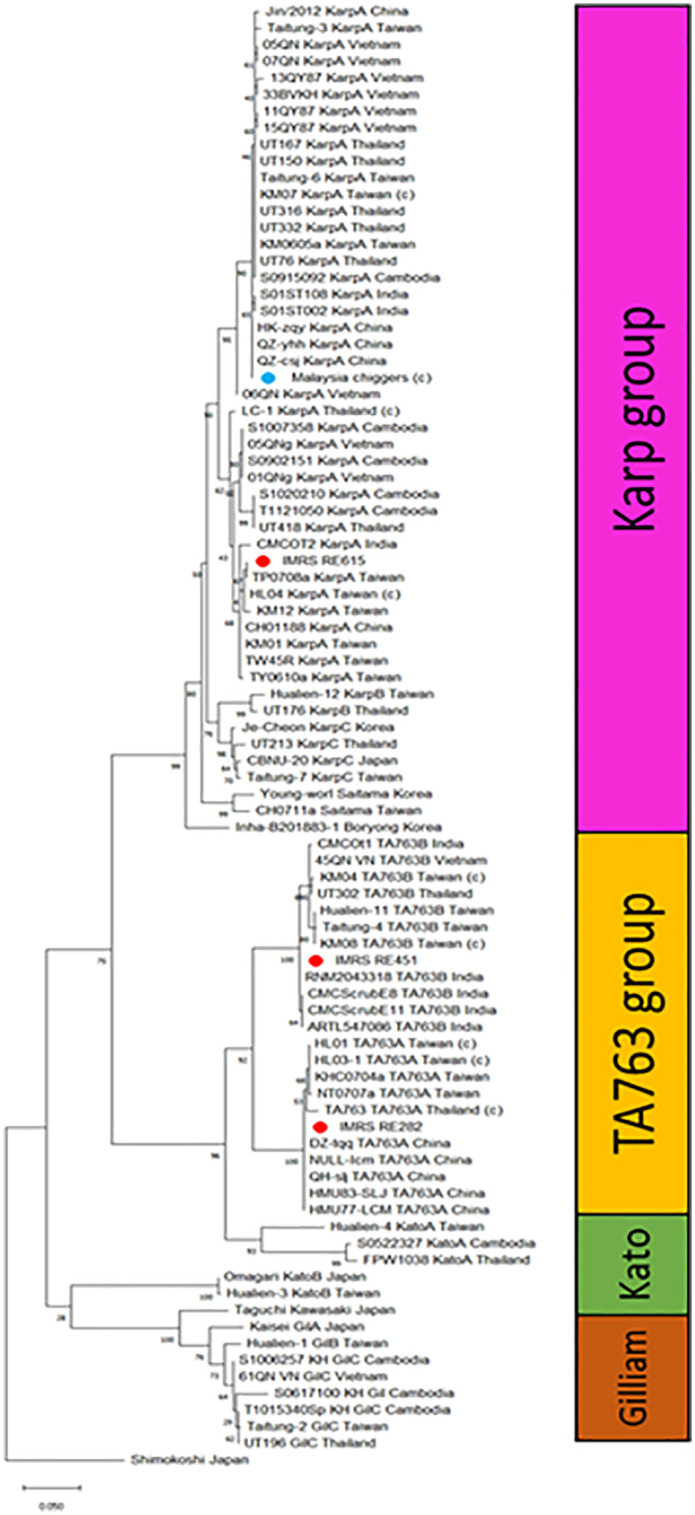
Phylogenetic tree showing the three fatal human cases RE282 (Case 1), RE451 (Case 2), RE615 (Case 3) and Msia chiggers (positive chigger case) labelled in red and blue, respectively. All four cases were analysed together with 84 *tsa56* sequences of various OT genotypes which includes Karp group (KarpA, B, C, Saitama, Boryong), TA763 (A and B) group, Kato group **(A, B)**, Gilliam group (GilA, GilB, GilC, Kawasaki), and Shimokoshi as the outlier. Both human and chiggers cases are included in the analysis. The chigger cases are denoted with **(c)**. The sequences used in this phylogenetic tree can be found in S1 Table.

List of sequences used for phylogenetic tree in Fig 4 as in S1 Table.

## Supporting information

S1 Table List of 88 *tsa*56 genes and their information used in this study. (XLSX)
